# Improved delivery of miR-1296 loaded cationic nanoliposomes for effective suppression of triple negative breast cancer

**DOI:** 10.1016/j.jsps.2021.04.007

**Published:** 2021-04-23

**Authors:** Lamyaa Albakr, Fulwah Yahya Alqahtani, Fadilah Sfouq Aleanizy, Abdullah Alomrani, Mohammad Badran, Hussein Alhindas, Futwan Al-Mohanna

**Affiliations:** a Department of Pharmaceutics, College of Pharmacy, King Saud University, Riyadh, Saudi Arabia; b Department of Cell Biology, King Faisal Specialist Hospital & Research Centre, Riyadh, Saudi Arabia

**Keywords:** miR-1296, Liposomes, Triple-negative breast cancer, Cellular uptake

## Abstract

Nowadays, microRNA is considered an attractive strategy for the effective treatment of cancer. A significant delivery of microRNA for cancer therapy remains a significant obstacle to target cancer cells. The restoring microRNA-1296 (miR-1296) has immense therapeutic efficacy in triple-negative breast cancer (TNBC). TNBC is an aggressive subtype of breast tumors with the progression of malignant transformation. This study aimed to develop a cationic nanoliposome that can serve as a miR-1296 carrier and studied its efficiency in TNBC. The efficacy of miR-1296 liposomes was evaluated on its apoptotic effect, cellular uptake, and potential chemotherapy sensitization in the TNBC cell line (MDA-MB-231). For *in vitro* viability study, the apoptotic effect was performed to validate protein expression using Alamar blue kit and western blot. The transfection of miR-1296 into TNBC cells was also investigated using cisplatin as a TNBC resistance drug. The fluorescent miR-1296-cy3 liposome was used for cellular uptake study. The miR-liposome was successfully prepared with a particle size of 123.6 ± 1.3 nm and encapsulation efficiency of 94.33%. A dose of 0.5 uM has significantly reduced the viability of MDA-MB-231 to be 33.45%±5.29 (P < 0.001). This result was validated by down-expression of CCND1, and PARP1, the miR-1296 receptor, and apoptosis marker. The image of the miR-1296-cy3 liposome showed cytoplasmic intracellular localization. It was found high sensitization of TNBC cell line for miR-1296 liposome compared to cisplatin (P < 0.001). Future *in vivo* research may answer questions concerning safety and stability. This study demonstrates that miR-191 liposomes may have promising clinical applications for TNBC therapy.

## 1 Introduction

Triple-negative breast cancer (TNBC) is characterized by lacking the expression of all three therapeutic targets, human epidermal growth factor receptor 2 (HER2), progesterone (PR), and estrogen (ER) (Ballinger et al., 2016). TNBC patients suffer from the poorest prognosis with a median overall survival rate of fewer than two years (O'sullivan et al., 2018). There is an urgent need to identify novel therapeutic entities to treat TNBC. Currently, there are quite promising new therapeutic lines for TNBC, including RNA interference (RNAi) therapeutics.

Several deregulated tumor-suppressive miRNAs with pivotal rules in TNBC were identified. For instance, miR-205 was found to be down-regulated in TNBC, and by the forced expression decreased cell proliferation *in vitro* and inhibited tumor growth *in vivo* (D'Ippolito & Iorio, 2013). Wang J *et al*. found that miR-206 expression is significantly reduced, and its replacement in TNBC cells would inhibit their cell migration (Wang et al., 2014). Other important deregulated tumor suppressor miRNAs include miR-200c (Humphries et al., 2014), miR-145 (Eades et al., 2014), miR-539 (Yang et al., 2018), and miR-340 (Shi et al., 2017). The fundamental role tumor-suppressor miRNAs play in TNBC has made them an interesting candidate in drug therapeutics development (Rupaimoole & Slack, 2017). Several studies were linked to their down-regulation with the development of different pathological cascades in TNBC, ranging from antineoplastic drug resistance, cell proliferation, development of metastasis, and were all demonstrated in miR-1296, which is the main subject of this work (Phan et al., 2016). Levels of such deregulated tumor suppressors miRNAs can be restored by the introduction of synthetic miRNAs (or miRNAs mimics) (Yin et al., 2014).

Functional studies have documented down-regulation of miR-1296 in different tumors, for example, prostate, cervical, gastric, ERBB2 positive, and TNBC (Chen et al., 2017, Leung et al., 2014, Majid et al., 2010, Phan et al., 2016, Shan et al., 2017). miR-1296 down-expression was associated with multiple tumorigenic mechanisms. Prior work demonstrated that re-expression of miR-1296 in such cancers would suppress tumor growth, block cell invasion and metastasis, induce tumor cell apoptosis, and interfere with cell cycle at different time point phases (Chen et al., 2017, Majid et al., 2010, Phan et al., 2016, Shan et al., 2017).

In TNBC, forced expression of miR-1296 inhibited cell proliferation, promoted cell apoptosis, and sensitize the cells toward platinum-based treatment (Phan et al., 2016). This suggested the role of miR-1296 as a vital tumor suppressor (Chen et al., 2017, Majid et al., 2010, Phan et al., 2016, Shan et al., 2017). However, these studies have used commercial transfection reagents in which the function and mechanism of miR-1296 re-expression were thoroughly studied (Chen et al., 2017, Majid et al., 2010, Phan et al., 2016, Shan et al., 2017). Naked miRNA delivery is a challenging task; this is due to their poor penetration (Huang et al., 2015), biological instability (Yu et al., 2009), short half-life *in vivo* (Chen et al., 2015), rapid clearance (Garzon et al., 2010). In brief, due to poor penetration, pharmacokinetics, and stability, miRNA requires delivery systems to efficiently enter tissues and cells (Deutsch et al., 1989). Ideal delivery systems allow the miRNA load safely to overcome physiological and cellular barriers. As previous studies showed, these barriers were overcome with superiority using different models of liposomal vectors (Shishir et al., 2019, Shishir et al., 2021). Cationic liposomes are widely used as gene vectors (Ozpolat et al., 2014). The remarkable progress achieved for cationic liposomes in gene therapy lies in the interaction that occurs between the positive charge of the liposomes with the negative phosphate group charge of the nucleic acid and with the outer cell membrane (Ozpolat et al., 2014, Pisani et al., 2011). In this study, we investigated the development of cationic nanoliposome delivery systems for miR-1296 and characterized their cellular uptake, *in vitro* viability, apoptosis, and potential chemotherapy sensitization.

## 2 Materials and methods

### 2.1 Materials

1,2-dioleoyl-3-trimethylammonium-propane (DOTAP) was obtained from Avanti Polar Lipids (Alabaster, Alabama, United States). Cholesterol (Cholesterin®) was obtained from Riedel-de-Haën™ (Honeywell, Germany). miR-1296 mimic (accession no. MIMAT0005794) and control miRNA (NC) were purchased from Life Technologies (Carlsbad, California, United States). Fluorescently labeled miR-1296-CY3 conjugated was purchased from Invitrogen (Carlsbad, California, United States). Quant-iT™ RiboGreen® RNA Assay Kit was purchased from Invitrogen (Carlsbad, California, United States). HaltTM Phosphatase Inhibitor Cocktail 100X and HaltTM Protease Inhibitor Cocktail 100X were purchased from Thermo Scientific (Waltham, Massachusetts, United States). All primary antibodies, including CCND1 (cat no. 2978S), PARP1 (cat no. 2532S), and housekeeping gene antibody GADPH (cat no. 5174S), were purchased from Cell Signalling (Massachusetts, United States). Horseradish Peroxidase (HRP) conjugated mouse IgG kappa binding protein (m-IgGκ BP, cat no. sc-516102) was obtained from Santa Cruz Biotechnology (Texas, United States).

### 2.2 Methods

#### 2.2.1 Preparation of nanoliposomes

Briefly, 3:1 M ratio of DOTAP to cholesterol were dissolved in 2:1 v/v chloroform: methanol. The lipid mixture was evaporated using a rotary evaporator (Büchi, Switzerland) under reduced pressure (258 mBar) and temperature above the phase transition temperature ($T_{m}$) of DOTAP (11.9 °C). The thin film was formed and then hydrated with trehalose (5% w/v) dissolved in nuclease-free water. This suspension was manually shaken to completely disperse the lipid film. After that, small unilamellar vesicles were obtained using Sonics Vibra Cell VCX-75 probe sonicator (Connecticut, New England) at 68% amplitude for 10 min under an ice bath. The blank nanoliposomes (NL) were filtered using a 0.45a μM syringe filter to remove titanium particles generated from the probe sonicator. The blank NL were stored at four ℃ until miR-1296 loading.

#### 2.2.2 MicroRNA loading

NL was incubated with an equal volume of miR-1296 at an N/P up to 1/10th under simple mixing and allowed to complex room temperature for 30 min to obtain NL-miR-1296. Moreover, the lyophilized blank nanoliposomes were also hydrated with RNase-free water containing miR-1296 at the desired N/P ratio up to 1/10th of initial pre-lyophilization volume (100 μL). Following,1X PBS was added to a volume that matches initial pre-lyophilization NL(500 μL).

#### 2.2.3 Measurement of vesicle size, polydispersity index, and zeta potential

Particle size distribution (z-average mean) and polydispersity index (PDI) were measured using dynamic light scattering (DLS) using a photon correlation spectrometer Brookhaven 90 Plus (New York, United States) as previously described (Hayward et al., 2016). The measurement was done in triplicate after dilution with 10 mM PBS, pH 7.4, and the results were averaged.

Zeta potential was measured as previously described (Sharma et al., 2017) using Malvern Zetasizer Nano-ZS (Worcestershire, United Kingdom). The obtained samples were measured in triplicate at room temperature after dilution with 10 mM PBS, pH 7.4. The average was calculated for each sample.

#### 2.2.4 Scanning electron microscopy (SEM)

The surface morphology of NL and NL-miR-1296 was assessed using SEM at an accelerating voltage of KV and a secondary detector. Freshly prepared samples were allowed to dry on a glass slide then examined using JEOL JSM − 6060LV (Tokyo, Japan).

#### 2.2.5 Transmission electron microscopy (TEM)

The phosphotungstic negative stain method was used for morphology assessment of nanoliposomes (NNL, NNL-miR-1296). Simply, a drop of each sample was applied to separate copper girds coated with carbon film and left to dry in the air. Samples were treated with 2% phosphotungstic acid solution and further analyzed using JEM-1400 Plus TEM from JEOL (Tokyo, Japan).

#### 2.2.6 Encapsulation efficiency (EE%)

The EE% of miR-1296 in prepared liposomes were determined by Quant-iT Ribogreen RNA kit as previously prescribed (Landesman-Milo et al., 2013). Briefly, the calibration curve of a reference ribosomal RNA (rRNA) standard was constructed at different concentrations (0, 500, 1500, 2500, 3500, and 4000 ng/mL) by dilution with Tris-EDTA buffer. Ribogreen RNA quantitation reagent was added to each sample and measured at excitation at 485 nm and emission at 525 nm. Background fluorescence was subtracted from the blank solution. Another calibration curve was constructed using the same miR-1296. The EE% was determined by comparing the fluorescence of RNA binding dye in the presence and absence of the 0.5% Triton X-100 in RNase free 1XPBS. In the absence of Triton X-100, fluorescence represents only free (unentrapped) miRNA as unloaded Ribogreen dye into nanoliposomes. In the case added Triton X-100, the fluorescence represents total miRNA (by adding the detergent followed by vortex agitation for 10 min). EE% was calculated as the following equation:

$$EE\%=1-\frac{Free\;miRNA\;concentration\;\left(without\;0.5\%\;Triton\right)}{Total\;miRNA\;concentration\;\left(with\;0.5\%\;Triton\right)}\times 100$$

#### 2.2.7 Gel retardation assay

Agarose gel (1–2%) was used to study the complexation of mir-1296 to NL at different N/P ratios. Agarose gel was stained with ethidium bromide and run for 40 min on 80 V. Images were visualized by Image Quant LAS 4000 (Sunnyvale, California).

#### 2.2.8 Serum stability assay

The stability of NL-miR-1296 against serum enzymes, including RNase A, was tested using 1% agarose gel obtained from MilliporeSigma (Burlington, Massachusetts) as previously described (Liu et al., 2016). NL-miR-1296 was mixed with fetal bovine serum (FBS) in a 1:1 ratio and incubated at 37° C. After 1, 3, and 24 h, aliquots from the mixture were further analyzed on 1% agarose containing ethidium bromide. The bands were visualized and compared to free miR-1296 exposed to the same conditions.

#### 2.2.9 In vitro release of miR-1296 loaded liposomes

The *in vitro* release profile was performed on NL-miR-1296 and free miR-1296 (equivalent to 0.017 mg of miRNA). An equivalent volume of the sample was dispersed in 4 mL of Tris-EDTA (TE) buffer of 7.4 at 37±1 °C and 200 rpm shaking. At predetermined time intervals, aliquots were withdrawn and replaced with an equal volume of fresh TE buffer. The number of released miR-1296 was determined using a specific RNA binding dye(Quant-iT Ribogreen) in the presence and absence of 0.5% Triton X-100 as described previously (Kapoor & Burgess, 2012). The RNA dye was not able to penetrate intact lipid; therefore, in the absence of Triton X-100, only the fluorescence of the released miR-1296 was recorded. Whereas, in the presence of the Triton X-100, total fluorescence was recorded to determine the % release profile at a time (t). The experiment was done in triplicate, and average±SEM was calculated.

#### 2.2.10 MDA-MB-231 cell line culturing

Human metastatic TNBC cell line (MDA-MB-231) were cultured in DMEM containing 4 mM/L-glutamate, 3.7 g/L sodium bicarbonate, 1 mM/L sodium pyruvate, 4.5 g/L glucose and supplemented with 10% FBS, and 1% antibiotic–antimycotic This media is termed as “complete media.” Cells were cultured at 37 °C in a humidified atmosphere with 5% CO_2_. For cell counting, cells were trypsinized, and the sample was withdrawn for counting using Scepter Cell Counter-top (Merck Millipore, Burlington, Massachusetts).

#### 2.2.11 Transfection of TNBC cell line

Cells were seeded in 24-well plates at a density of 4 $\times 10^{5}$ cells/mL (2 $\times 10^{5}$ Cells/well). After 24 h, transfection of miR-1296 or its negative control was performed using transfection reagent Lipofectamine 3000 (Life Technologies, Carlsbad, California). Before transfection, the medium was changed to serum-free DMEM media, and cells were treated using different concentrations of miR-1296 loaded liposomes and its negative control (0.1–3 μ M). After 5 or 18 h of exposure, miRNA complexes and reagents were removed, and media was replaced with complete media.

#### 2.2.12 Cytotoxicity assay

Cell cytotoxicity was performed using Alamar Blue (Resazurin) redox viability assay (BioRad, Hercules, California). MDA-MB-231 cells were seeded at a density of $1.5\times 10^{5}$ cells/well in a 24 well plate. When cells were 70% confluent (or after 24 h), media was replaced with serum-free DMEM media, and cells were treated with 50 μL of NL-miR-1296 or NL-miR-NC forfive5 h. After that, cells were washed with 1XPBS, and the media was changed to a complete medium.

Alamar Blue reagent was added to a volume of 10% of each well. After 2 h, fluorescence was recorded at 550 excitations and 590 emission wavelengths using SpectraMax M5 (Moleculardevices, San Jose, California). The viability of cells was expressed relative to untreated cells viability (%) as follows:

$$\%\;Cell\;viability=\frac{Fluorescence\;of\;treated\;cells}{Fluorescence\;of\;untreated\;cells}\times 100$$

#### 2.2.13 Nanoliposome safety study

Cytotoxicity of cationic nanoliposomes was tested by treating MDA-MB-231 with different N/P ratios of NL-miR-1296 and NL-miR-NC alone for 5 h, and the media changed after that. Cytotoxicity was evaluated using the Alamar Blue kit as described in the previous section.

#### 2.2.14 Cell fixation and nuclei staining

Firstly, the cells were rinsed with pre-warmed 1XPBS twice. Fixation was done using freshly prepared 3.7% paraformaldehyde for 15 min at room temperature. Cells were further washed with 1XPBS for 10 min. The staining of the nuclei was performed for 3 min using DAPI at 1:1000 dilution in 1XPBS-0.5%BSA. Cells were covered and protected from light for further visualization using a fluorescence microscope.

#### 2.2.15 Intracellular uptake study

Cells were seeded on coverslips using 6-well plates ($1\times 10^{5}$ cells/well). On the following day, cells were treated with 0.5μ M of NL-miR-1296-cy3. Cells were fixed at 2, 4, 6, and 24 h post-treatment. Images were taken using upright automated fluorescent microscope BX63 (Olympus, Tokyo, Japan).

#### 2.2.16 Protein extraction from cell line

MDA-MB-231 cells were seeded in 60 mm cell culture dishes (1$\times 10^{5}$ Cells). At 70% confluency, cells were treated with 0.5μM (250 pmol) of NL-miR-1296 or NL-miR-NC for 5 hrs. After 5 hrs, media was changed by serum-containing DMEM. Cells were harvested 48 h post-treatment and further lysed using radioimmunoprecipitation assay (RIPA) buffer combined with Halt phosphatase and Protease Inhibitor Cocktail 100X obtained (Invitrogen, Carlsbad, California) and kept on ice for 20 min. Protein was collected from the supernatant after centrifugation at 14,000 rpm for 15 min in a pre-cooled centrifuge and further kept in $(-30^{{}^{\circ}}C$) for further analysis. Each sample was done in triplicate.

#### 2.2.17 Protein quantification

The total protein extracted from the cells was quantified using Direct Detect Spectrometer from Millipore (Burlington, Massachusetts). About two μL of protein lysate loaded on the Direct Detect assay-free sample card, which contains four hydrophilic polytetrafluoroethylenes (PTFE) membrane positions surrounded by a hydrophobic ring to retain the analyzed sample within the device's Infrared (IR) beam. Sample concentrations were determined in reference to the built-in bovine serum albumin (BSA) calibration curve. Ten concentration points of BSA were diluted in PBS (0.125–5μ g/μL) were used to generate a robust instrument calibration curve (Cappione et al., 2012, do Carmo et al., 2016). RIPA buffer was analyzed as a reference blank. An average of three measurements was considered.

#### 2.2.18 Protein blotting

Samples were diluted with RIPA buffer to reach an equal protein amount in each well (55μ g, total volume = 15 μL). Samples were then mixed with equal volumes of 2X Laemmli buffer, containing 5% β-mercaptoethanol as a reducing agent, and heated on thermomixer at 99 °C for 5 min. The samples were then loaded into SDS-PAGE, any kD precast polyacrylamide gel along with a standard protein marker and electrophoresed for 5 min at 50 V in 1X running buffer (Tris/Glycine/SDS Buffer). The voltage was increased to 150 V for 45 min.

Proteins were then transferred from gel to 0.2μM PVDF membrane using the Trans-Blot Turbo transfer system for 3 min. Non-specific binding to membranes was blocked by incubation of the membrane in Tris-buffered saline-tween (TBS-T) with 5% w/v skimmed milk at room temperature on a shaker for 2 hrs. Blots were washed after blocking two times in TBST every 5 min and then in TBS for 5 min. After that, membranes were probed with indicated primary antibodies diluted with blocking buffer (1:1000) overnight with agitation at 4 °C.

The next day, membranes were washed two times for five minutes with TBST and one time with TBS for 5 min on a shaker. Horseradish Peroxidase (HRP) was a conjugated secondary antibody (IgG) that is diluted 1:1000 in 5% skimmed milk-TBS. Membranes were incubated in the secondary antibody solution at room temperature on a shaker for 1 h. Membranes were then washed three times each for 5 min in TBST. The enhanced chemiluminescent (ECL) substrate solution was added to membranes according to manufacturer protocol; about 3.5 mL from peroxide solution and 3.5 mL were added from luminol enhancer solution and incubated for 5 min on a shaker in a dark room and protected from light. Visualization of the band was performed using the ECL detection CCD camera system from BioRad (Hercules, California). GADPH was used as an internal control housekeeping gene.

#### 2.2.19 Chemotherapy sensitization study

The sensitivity of TNBC cell lines to cisplatin has been reported and enhanced by miR-1296 treatment (Phan et al., 2016). To study the effect of NL-miR-1296 on sensitizing of MDA-MB-231 to the chemotherapeutic agent, cells were treated with NL-miR-1296 or NL-miR-NC. After that, cells were treated with different cisplatin concentrations (0.5 to 100μ M). Then, 48 h -post-transfection, the viability of the cells was assessed using Alamar blue assay.

#### 2.2.20 Stability assay

The stability of NL-miR-1296 was evaluated at different possible proper storage temperatures (25 °C, 4 °C, −30 °C, and −80 °C) for 80 days. At predetermined time points (0, 40, and 80 days), samples were tested as a function of particle size (nm), polydispersity index, and zeta potential (mV).

Biological testing was done to assure the functionality of NL-miR-1296 at different storage conditions and time points. After 40 or 80 days, MDA-MB-231 cells were seeded at a density of $1.5\times 10^{5}$ cell/well in a 24 well plate. When cells were 70% confluent (or after 24 h), media was replaced with serum-free DMEM media, and cells were treated with 50 μL of NL-miR-1296 that previously-stored at different temperatures. Media was changed into complete media (DMEM 10% FBS, and 1% AB) after 5 hrs. The viability of the cells was tested after 48 h using Alamar Blue.

Samples were kept at room temperature at least 30 min before measurement. All measurements were done in triplicate, and data represented as average ± SEM.

#### 2.2.21 Statistical analysis

Differences between experimental groups were evaluated using unpaired two-tailed Student's *t*-test. A p-value of ≤0.05 is considered significant. One-way analysis of variance (ANOVA) followed by Tukey's multiple comparisons test was performed when appropriate (GraphPad Prism version 8.0.0 for macOS Software, Inc). Results were expressed as averages± SEM.

## 3 Results

### 3.1 Characterization of NL-miR-1296

**Fig. 1:**
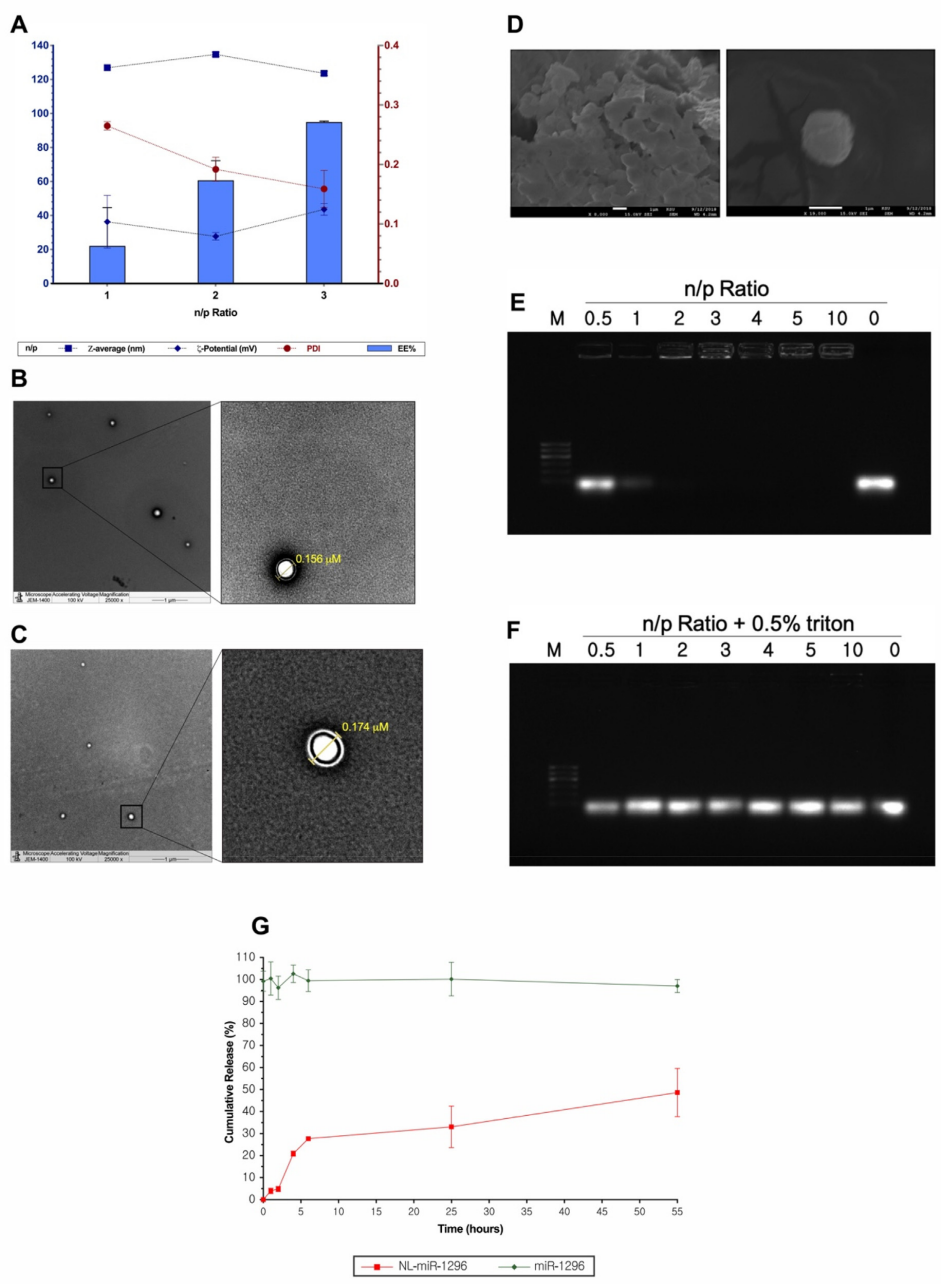
Characterization of NL-miR-1296. (A) Particle size, PDI, zeta potential and EE% characterization of NL-miR-1296. (B) Transmission electron microscopy of plain NL, and NL-miR-1296 (C) TEM images. (D) present scanning electron microscope overview image of prepared NL-miR-1296; (E) 1% agarose gel used to test the complexation of miR-1296 with NL at different n/p ratios. Different n/p ratios used 0.5–10, where 0 represent free miR-1296 only. All wells contain the same amount of miR-1296 (0.65 μg) but various NL part. The same n/p ratios were used but with NL were permeabilized with 0.5% triton. NL-miR-1296 were allowed 30 min to complex before running. Run was for 40 min at 80 V using TBE buffer. Ethidium bromide (0.5 μg/mL) was used to stain the gel; (F) Characterization of fresh NL-miR-1296 with different n/p ratios. Data represent mean ± SEM; (G) In vitro release of miR-1296 from NL-miR-1296. The release was evaluated in pH 7.4 using TE buffer, at 37 °C. Free miR-1296 was tested for integrity purposes. Points represent averages ± SEM.

Here, NL-miR-1296 was developed for the evaluation of anti-miR-191 delivery in TBST. The NL-miR-1296 formulations were characterized for their mean size distribution, surface charge, and vesicle morphology, and EE% depending on different N/P ratios of NL-miR-1296 (Fig. 1A). NL-miR-1296 were successfully obtained at an average particle size of 126 nm ± 1.4, 134.7 nm ± 1.4, and 123.6 nm ± 1.3 at different n/p ratio. The positive zeta potential values of NL-miR-1296 were dependent on the incorporation of DOTAP.

The NL-miR-1296 and plain NL showed spherical morphology with predominantly unilamellar vesicles (Fig. 1 B and C, respectively). SEM images further confirmed the spherical shape of NL-miR-1296 (Fig. 1D).

Both fresh and lyophilized NL were characterized for miR-1296 encapsulation (Fig. S1). At all n/p ratios, fresh NL-miR-1296 showed higher efficient loading of miR-1296 (Fig. S2). the EE% increased dramatically from 22% to 94.47% when the n/p ratio increased from 1 to 3 (Fig. 1A). Thus, anti-miR-1296 is strongly adsorbed on the NL surface, making a stable complex. For this reason, fresh NL-miR-1296 was further used in biological and stability analysis. Moreover, the complexation of miR-1296 with NL at different N/P ratios was assessed using 1% agarose gel. About 0.65 μg of miR-1296 was used to prepare all complexes (but nitrogen ratio vary from 1 to 3). As shown in Fig. 1E, complexation started at the n/p ratio of 1. However, complete complexation was obtained at the n/p ratio of 3. About 0.5% Triton was used to lyse the liposomes and free their miR at all n/p ratio to confirm the equal quantity of loaded miR-1296 in all wells, including free miR-1296 (Fig. 1F).

In vitro miR-1296 release profile form NL-miR-1296 (n/p ratio of 3) was illustrated in Fig. 1G. About one-third of the miR-1296 amount loaded in NL was released on the first day. Thus, the cumulative release rate of miR-1296 throughout 24 h in TE buffer (pH = 7.4) was 33.45 ± 6.57%. The amount released of miR-1296 was gradually increased throughout the next 24 h. To ensure the integrity of miR-1296 throughout the experiment selected condition (pH = 7.4, 37 °C), an experiment with the same amount of naked miR-1296 was run and analyzed at the same time (Fig. 1G). As shown in Fig. 1G, the naked miR-1296 totally degraded (no bands).

### 3.2 Intracellular uptake of NL-miR-1296

**Fig. 2:**
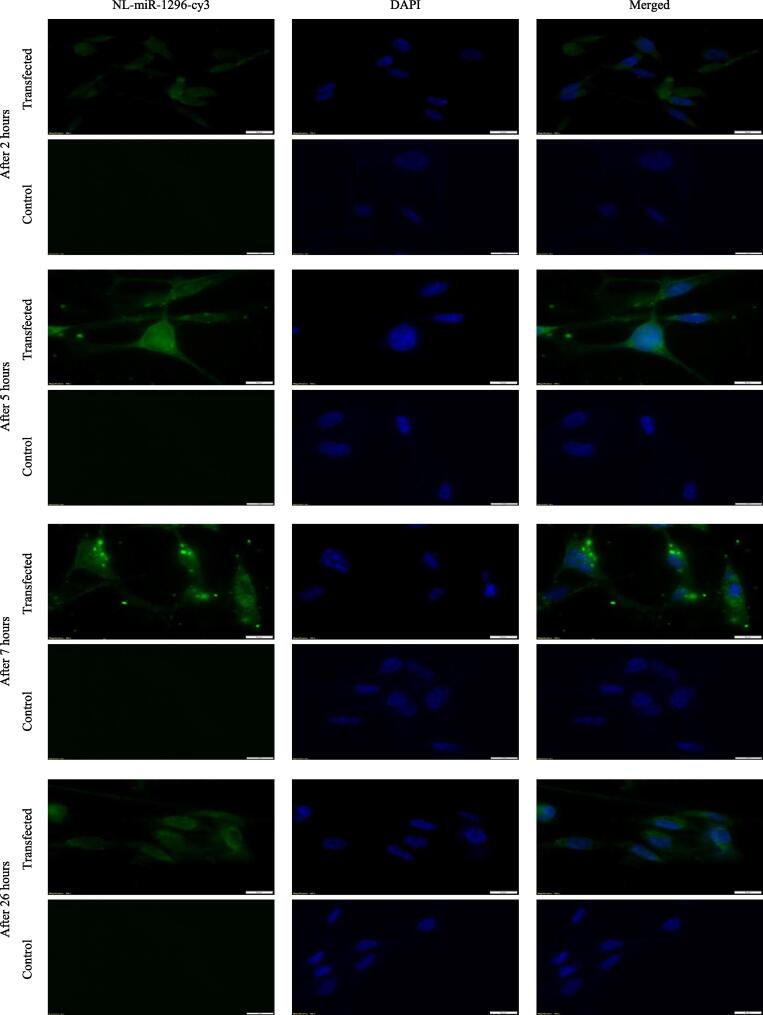
Intracellular uptake of NL-miR-1296-cy3 in MDA-MB-231. About 100 thousand cells were seeded in coverslips in a 6-well cell culture plate. Next day, cells were treated with NL-miR-1296-cy3 in serum-free media (green). After 2,5,7, and 26 h, coverslips were fixed using 3.7% formaldehyde. Nuclei stained with DAPI (blue). Scale bars = 10 µm.

As clear in Fig. 2, in almost all time points, cells appeared with green cy3 uptake in the cytoplasm (miR-1296 site of action). However, after 5 and 7 h of treatment, MD-MB-231 cells showed apparent high green fluorescence, suggesting a full, robust uptake of miR-1296 after 5 h. After 26 h, cells were of a clumpy appearance, suggesting the start of the apoptotic cascade.

### 3.3 MDA-MB-231 viability after treatment with NL-miR-1296

**Fig. 3:**
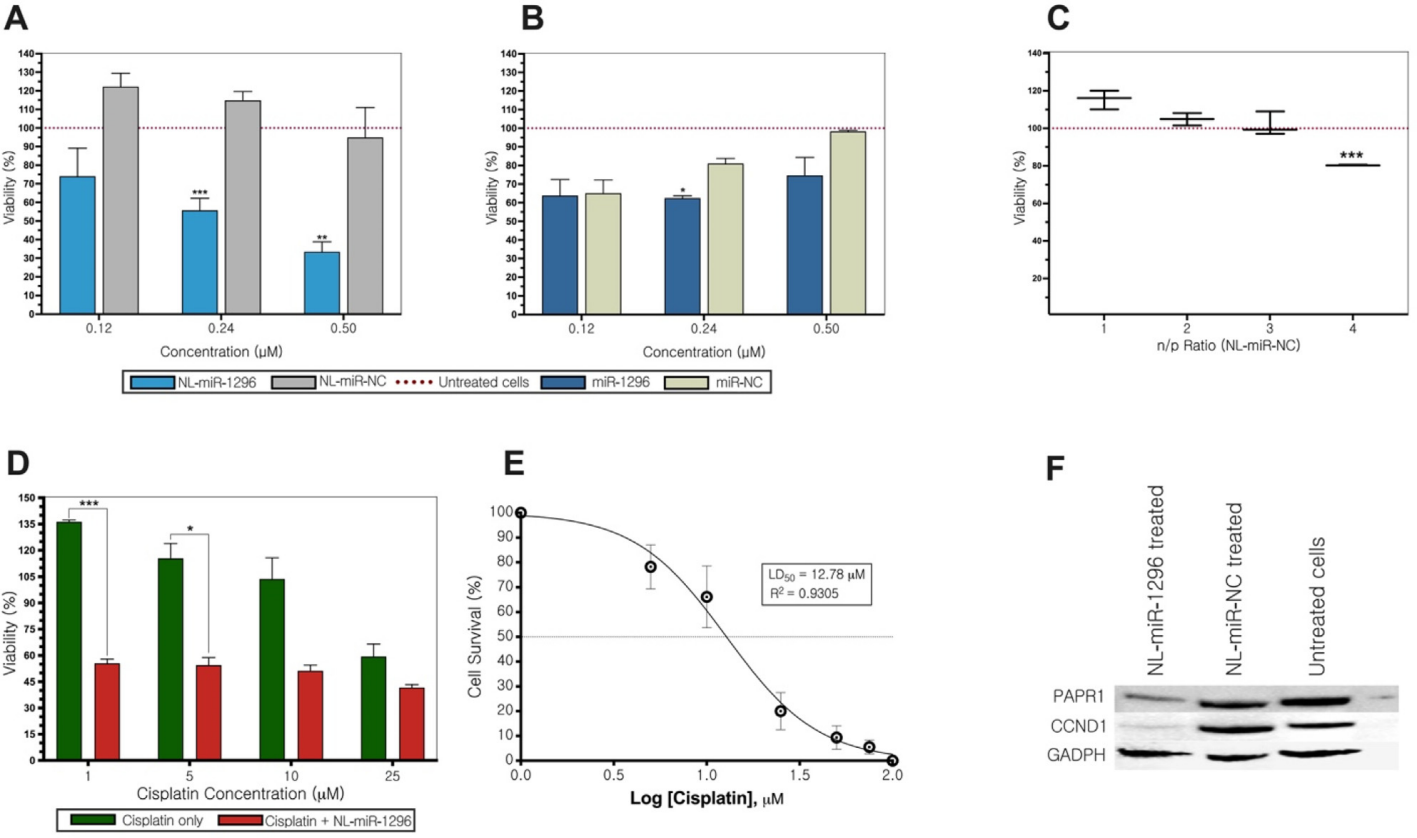
. Viability, chemotherapy-sensitization, and stability analysis of NL-miR-1296. (A) MDA-MB-231 viability after treatment with different concentrations (0.12–3 μM) of NL-miR-1296, or NL-miR-NC (B) Cells transfected using Lipofectamine. (C) Safety of nanoliposomes was analyzed using different n/p ratios (D) NL-miR-1296 sensitizes MDA-MB-231 to cisplatin treatment at a dose of as low as 1 μM. (E) A dose–response curve of cisplatin treatment in MDA-MB-231 cells (D) Down-expression of CCND1, and PARP1 in NL-miR-1296 treated MDA-MB-231 cells.

The transfection of the tumor suppressor miR-1296 could decrease MDA-MB-231 viability *in vitro* (Phan et al., 2016). 0.24 μM of NL-miR-1296 decreased the viability of MDA-MB-231 significantly compared to untreated cells (P < 0.001) and 0.5 μM (33.45 ± 5.29) Fig. 3A. By comparison with NL-miR-NC, the viability of MDA-MB-231 treated with NL-miR-1296 was significantly reduced to 0.25 μM (P < 0.001) and 0.5 μM (P < 0.002). However, the 0.5 μM dose decreased the viability to 33.45 ± 5.29 compared to 94.94 ± 14.68 of NL-miR-NC (Fig. 3A).

Fig. 3B showed the viability of MDA-MB-231 using different concentrations of miR-1296 by using a commercial transfection agent (Lipofectamine 3000). None of the indicated doses (0.12–3 μM) showed a significant decrease in viability compared to untreated cells. By using miR-NC, only doses of 0.24 μM and 1 μM showed a significant difference in viability compared to miR-1296 transfected (Fig. 3B). The safety of NL loaded with miRNAs on the viability of MDA-MB-231 was examined at the beginning exhibited no significant reduction of cell viability compared to untreated cells in n/p ratio from 1 to 3 (Fig. 3C).

### 3.4 NL-miR-1296 sensitize MDA-MB-231 to cisplatin chemotherapy

In the beginning, the dose–response curve was constructed for MDA-MB-231 treated with different concentrations of cisplatin alone (Fig. 3D). It has been shown that prior transfection with 0.5μ M NL-miR-1296 marked a reduction in the viability of MDA-MB-231 followed with dose 1μ M cisplatin (P < 0.001), and 5μ M (P ≤ 0.05) (Fig. 3E).

### 3.5 NL-miR-1296 decrease CCND1, and PARP1 expression

Protein expression analysis showed that NL-miR-1296 was able to decrease the expression of oncogenic CCND1 in TNBC cell line MDA-MB-231 compared to NL-miR-1296 NC or untreated cells (Fig. 3F). Also, TNBC was characterized by aggressive nature and resistance to apoptosis. In this regard, the expression of PARP1 (an apoptotic marker) was found to decrease when MDA-MB-231 was treated with 0.5 μM NL-miR-1296 mimics (Fig. 3F).

### 3.6 Effect of serum, storage temperature, and time on prepared NL-miR-1296

**Fig. 4:**
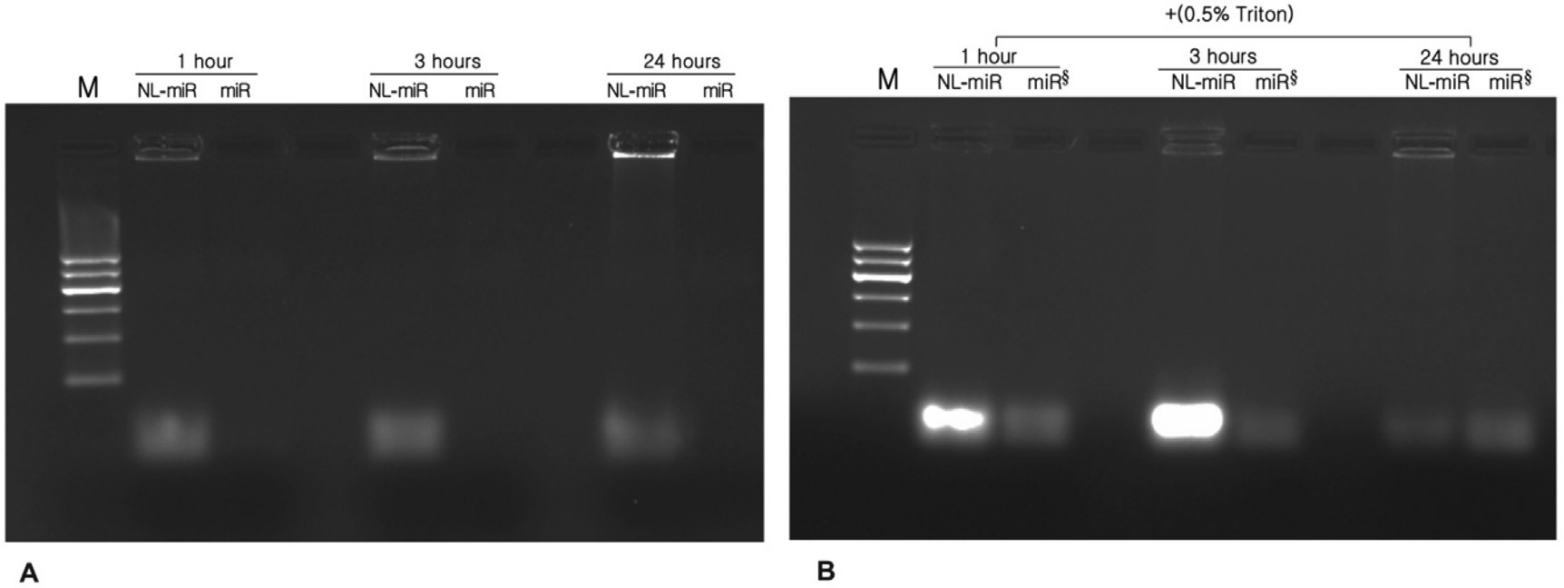
Effect of serum, temperature and time on NL-miR-231 stability. Serum stability was tested using 1% agarose gel. (A) simply samples of NL-miR-1296 (n/p ratio of 3) or free miR-1296 was incubated in 1:1 v/v of FBS (final concentration 50%) at 37 °C for 1, 3, and 24 h. (B) samples of the same NL-miR-1296 incubated in FBS for different periods were treated with 0.5%TritonX-100 just before the run and presented next to free miR incubated at the same time and temperature without FBS (denoted by §).

An equal volume of NL-miR-1296 was incubated with FBS to get a final concentration of 50% at 37 °C for 1, 3, and 24 hrs. No bands of free miR-1296 incubated with FBS at different time points were detected, revealing miR-1296 degradation. On the other hand, bright bands were detected in NL-miR-1296. The incubated NL-miR-1296 with FBS at different time points was treated with Triton X-100 (0.5% v/v) before loading to release, and subsequently, the miR-1296 loaded inside complexes were determined (Fig. 4A). After adding 0.5% Triton X-100, the bands of incubated NL-miR-1296 at different time points were brighter than free incubated miR-1296 without serum (Fig. 4B). From this, cationic NL at an n/p ratio of 3 could effectively protect miR-1296 from degradation by ribonucleases.

**Fig. 5:**
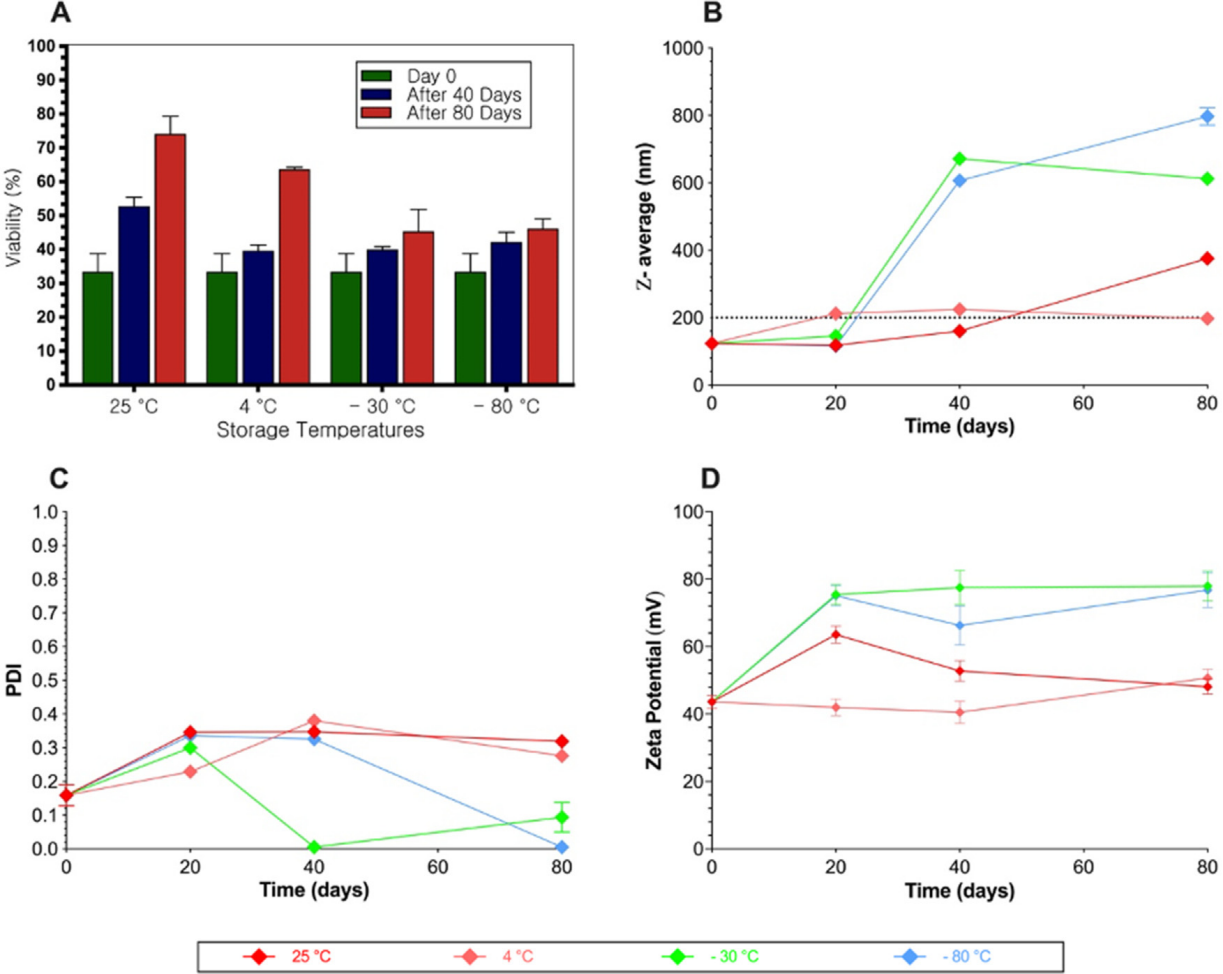
Effect of storage time and temperature on physical and biological activity of NL-miR-1296. (A) Interplay between stability and biological activity of NL-miR-1296 on TNBC at different time and storage temperatures. MDA-MB-231 cells were seeded in 24-well plate for 24 h. After 40 or 80 days, cells were transfected with NL-miR-1296 stored at different temperatures. Media was changed after 5 h, and viability was tested using Alamar Blue after 48 h and compared to untreated cells. Freshly prepared NL-miR-1296 was used transfect the cells as described above to represent 0-time point. Physical characterization of NL-miR-1296 stored in different temperatures at different time points was presented as (B) particle size, (C) PDI, and (D) zeta potential. Data represent the mean ± SEM of two independent studies (n = 6). (Multiple student *t*-test).

The biological activity of the stored NL-miR-1296 at different temperatures on the viability of MDA-MB-231 was evaluated and compared to freshly prepared NL-miR-1296 (Fig. 5A). After 40 days, there was no significant change in biological activity compared to freshly prepared NL-miR-1296. However, after 80 days, formulations were kept at 25 °C and 4 °C, which showed significantly less cytotoxic activity, P-value < 0.002, and < 0.05, respectively (Fig. 5A).

The physical appearance of the stored NL-miR-1296 at 25 °C, 4 °C, −30 °C, and −80 °C for 20, 40, and 80 days were evaluated. Particle size, PDI, and zeta potential of the kept formulation were stable for 80 days (Fig. 5B, C, and D). The particle size of stored NL-miR-1296 was maintained within the goal under 200 nm (Fig. 5B). Where the values of zeta potential showed no significant change throughout 20, 40, and 80 days compared to the fresh formulation (P-value = 0.64, 0.468, and 0.095, respectively), Fig. 5C. After Storage at −30 and −80 °C, the preserved biological activity (Fig. 5A) and the physical characteristic of NL-miR-1296 were changed dramatically (Fig. 5B, C, and D). The zeta potential(indicator in the stability of colloids) was increased to double at −30 and −80 °C after 20 days (Fig. 5D). On the other hand, particle size was started to increase after 40 days at −30 °C or less (Fig. 5B).

## 4 Discussion

It is commonly known that the use of miRNA contributes to the development of a new strategy for cancer therapy. Recently, miR-1296 was identified as an attractive approach for TNBC treatment. The main obstacle for the development of miR-1296 -based therapy is the successful delivery to its target tissue. Therefore, the established NL- miR-1296 using cationic lipids may effectively treat TNBC with a high loading of miR-1296.

Results from our study report high encapsulation efficiency of miR-1296 (above 90%) achieved using fresh cationic nanoliposomes in an n/p ratio of 3. In addition, In virtually all time points, cell efficiency uptakes the NL-miR-1296-cy3. After 7 h of treatment, higher green fluorescent intensity reported, suggesting a full, robust uptake of miR-1296 after 5 and 7 h. After 26 h, cells were of a clumpy appearance, suggesting the start of an apoptotic cascade.

It has been reported that miRNA bound to the cationic lipid of liposomes via electrostatic charge coupling led to an increase in cellular uptake (Yi Xue et al., 2015).

The high loading of miR-1296 was confirmed by a gel retardation assay at an n/p ratio of 3 by way of no migrated free miR-1296 band was detected. Our findings are consistent with cationic NL made of DOTAP:chol, that an n/p ratio of 3 showed complete retardation of miR-145 (Tao et al., 2016).

The release of free miR-1296 from NL-miR-1296 in serum stability study and not in the gel retardation assay, although the same n/p ratio used (3), can be explained by three reasons all attribute to physiological conditions. In the serum stability study, the NL-miR-1296 was exposed to the physiological pH (7), a temperature of 37 °C, and most importantly, to anionic serum proteins. All three conditions are believed to induce miRNA release from cationic nanoliposomes (Arora et al., 2014, Haghiralsadat et al., 2018). Moreover, faster release of miRNA in the presence of serum (>65%) compared to < 30% in the absence of serum due to polyanions in serum may compete with the anionic miRNA in binding to the cationic delivery system (Arora et al., 2014).

Previous work reported that IC50 of cells by using 50 μM of miR-1296 transfected by Lipofectamine 200 (Phan et al., 2016). But, our study revealed that 3 μM could reduce the viability of TNBC cell line significantly to 49% when compared to miR-NC 101% (P < 0.002) by transfection of miR-1296 to cells using Lipofectamine 3000.

Furthermore, the sensitization of MDA-MB-231 to cisplatin treatment after transfection with NL-miR-1296 was fairly comparable to previous literature (Phan et al., 2016). The NL-miR-1296 treatment can significantly decrease cell viability compared to cisplatin alone. A dose of 1 μM of cisplatin was sufficient for a significant decrease in viability of transfected cells (55%) when compared to the viability of cisplatin alone. In conclusion, the current study reported that NL-miR-1296 is considered a promising novel cancer therapy that acts on TNBC cell proliferation, apoptosis, and chemotherapy sensitization. Future *in vivo* studies may answer questions concerning safety and stability.

## Declaration of Competing Interest

The authors declare that they have no known competing financial interests or personal relationships that could have appeared to influence the work reported in this paper.
