# Supplementary material for: Improved delivery of miR-1296 loaded cationic nanoliposomes for effective suppression of triple negative breast cancer
Source: Saudi Pharm J. 2021 Apr 23;29(5):446–55. doi: 10.1016/j.jsps.2021.04.007 (PMC8180610; doi:10.1016/j.jsps.2021.04.007)
Supplement: Supplementary data 1 [file mmc1.docx]

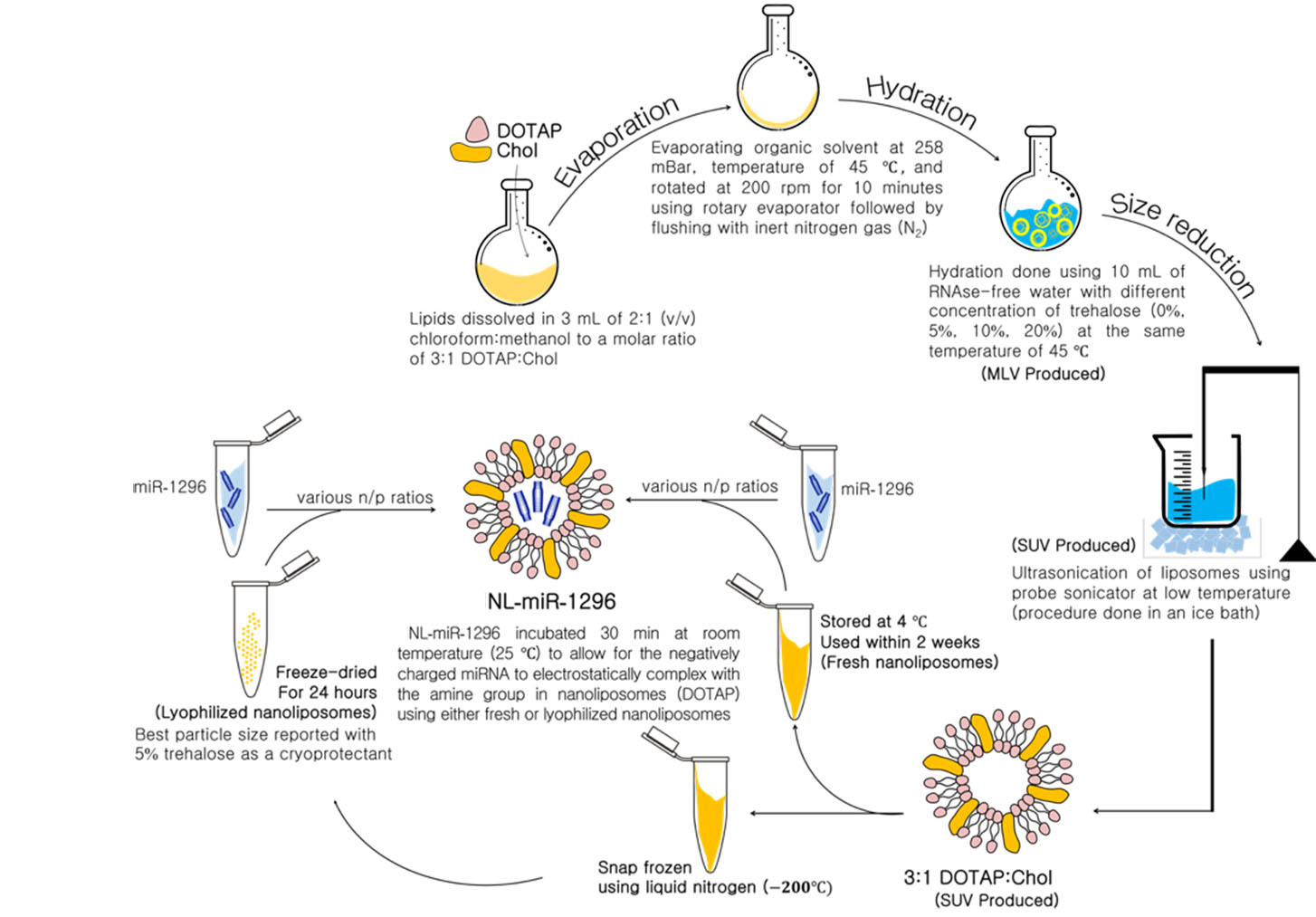


Figure S 1 Schematic representation of steps describing the production of cationic nanoliposomes loaded with miR-1296 (NL-miR-1296). Nanoliposomes prepared using thin film hydration method by dissolving 3:1 molar ratio of DOTAP: Chol in an organic solvent and further evaporating it. Hydration was done using different trehalose concentrations. Ultrasonication applied and successful production of SUV nanoliposomes. Both lyophilized and fresh nanoliposomes were considered for further analysis. Loading of miR-1296 was done using different nitrogen to phosphate ratio (n/p ratio) using either lyophilized or fresh nanoliposomes.


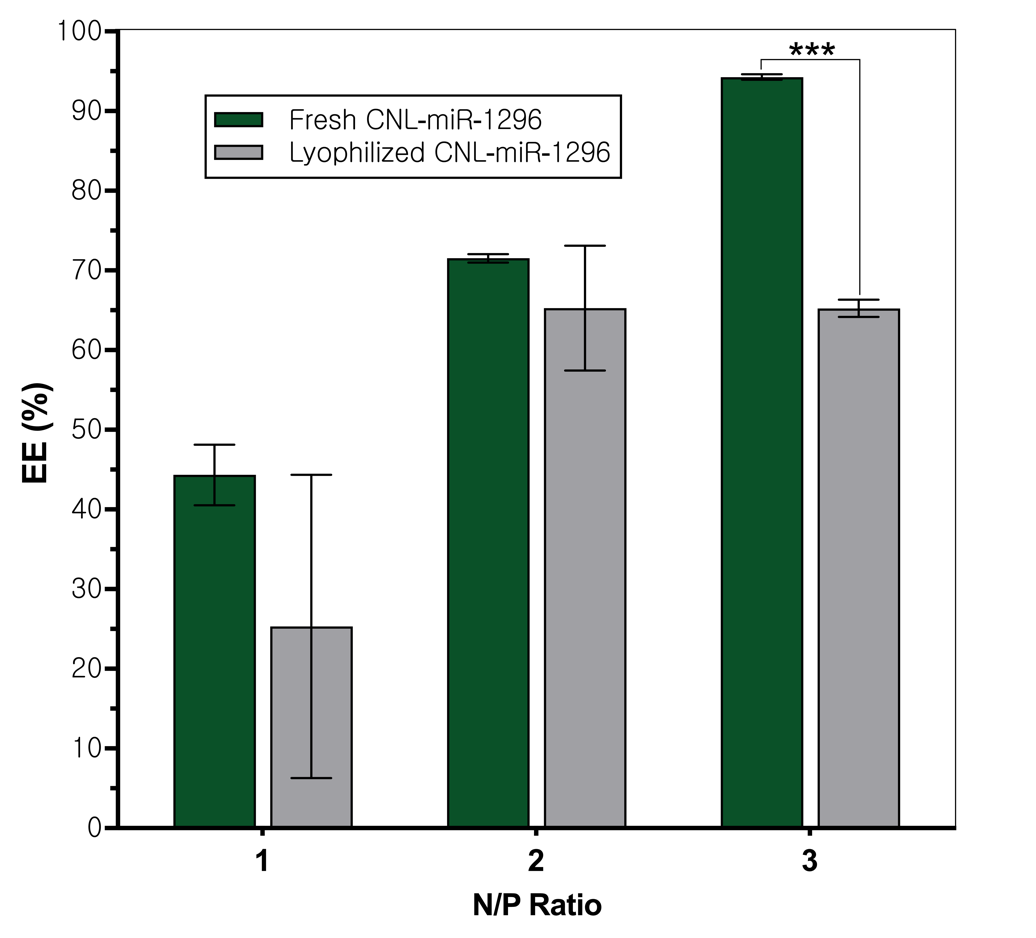


Figure S 2 EE% of miR-1296 loaded fresh versus lyophilized cationic nanoliposomes. Plain nanoliposomes were aliquoted, snap frozen, and further lyophilized before loading with miR-1296. EE% was determined using Ribogreen assay in triplicate, and average$\boldsymbol{\pm}$SEM was represented. ***$\boldsymbol{P}$<0.001.
